# Integrated full-scale solar CPC/UV-LED–filtration system as a tertiary treatment in a conventional WWTP for agricultural reuse purposes

**DOI:** 10.1007/s43630-022-00342-9

**Published:** 2022-11-19

**Authors:** L. Ponce-Robles, E. Mena, S. Diaz, A. Pagán-Muñoz, A. J. Lara-Guillén, I. Fellahi, J. J. Alarcón

**Affiliations:** 1 Department of Irrigation, Centro de Edafología y Biología Aplicada del Segura, CEBAS-CSIC, Murcia, Spain; 2 Municipal Water and Sanitation Company of Murcia (EMUASA), Murcia, Spain; 3 AQUAMBIENTE Services for the Water Sector, S.A.U., Madrid, Spain; 4 Technology Centre for Energy and the Environment (CETENMA), Murcia, Spain; 5 Mediterranean Agronomic Institute of Zaragoza (IAMZ)-CIHEAM, Zaragoza, Spain

## Abstract

Today, the emergence of increasingly restrictive treatment and reuse policies make the implementation of full-scale tertiary treatment, capable of improving the quality of water, a priority. Full-scale TiO_2_ photocatalysis systems are resulting in a promising option, since TiO_2_ is commercially available. However, questions such as how to work continuously during day/night irradiation cycle, or the removing of TiO_2_ in outlet flow are still unresolved. In this work, a full-scale system integrating a solar CPC/UV-LED step combined with commercial microfiltration membranes was installed in a conventional WWTP for agricultural reuse purposes. After optimization, 0.5 g/L of catalyst and combined SOLAR + UV-LED showing the highest pharmaceutical removal percentages, while a self-designed UV-LED included in the own reaction tank resulting in higher efficiencies compared with commercial lamps. Longer membrane surface area decreased fouling problems in the system. However, 60 min of irradiation time was necessary to reach the most restrictive water quality values according with (EU 2020/741). After optimization step, total costs were reduced by 45%. However, it was shown that a reduction in operating and maintenance costs, along with the development of more effective and economical commercial filtration membranes is a key factor; therefore, working on these aspects is essential in the treated water cost reduction.

**Graphical abstract:**

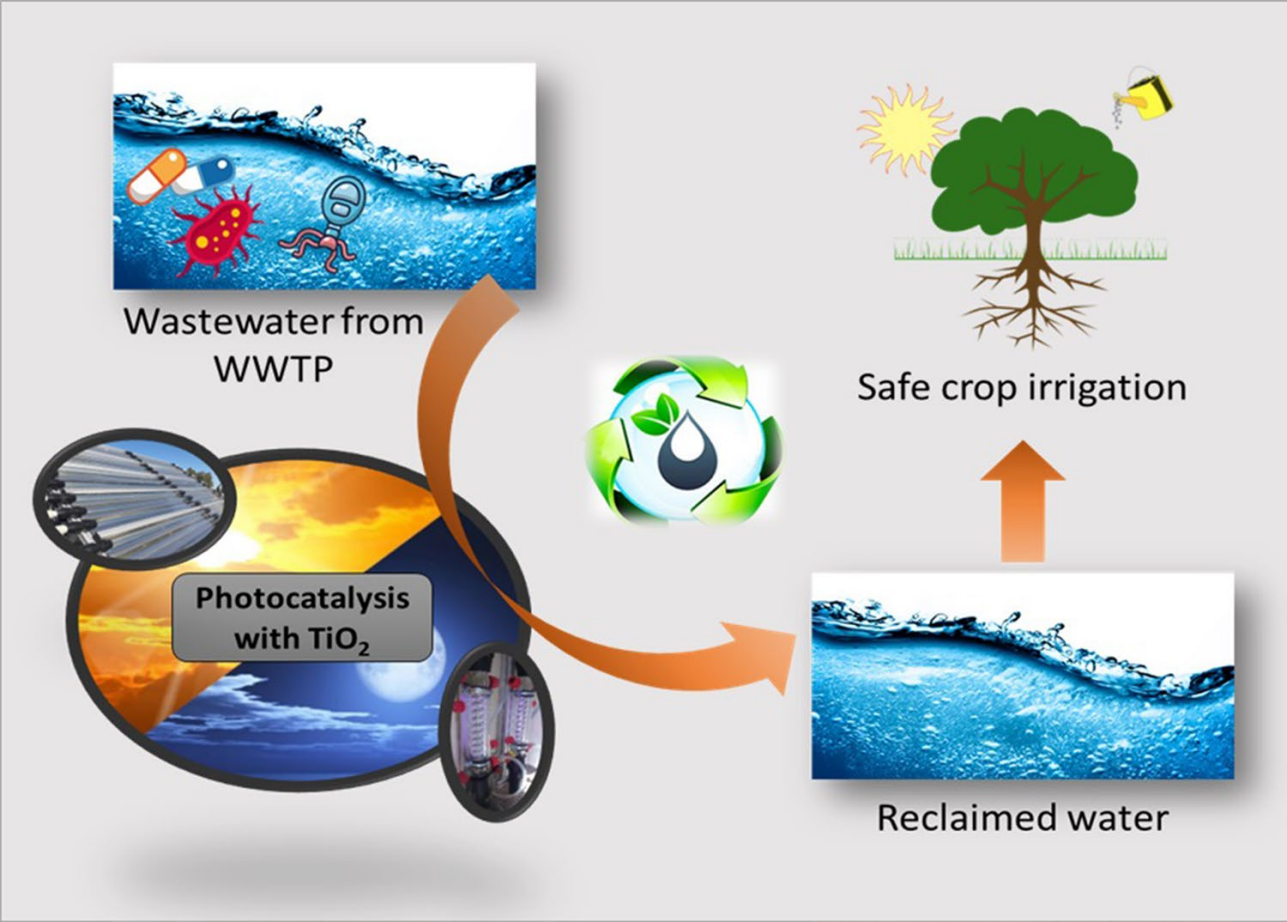

**Supplementary Information:**

The online version contains supplementary material available at 10.1007/s43630-022-00342-9.

## Introduction

The need for alternative water resources has been accelerated in recent years. By 2030, the world will face a global water deficit of 40% [1], and this situation is expected to worsen in the coming years due to population growth, climate change, and other indirect factors, such as economic problems and uncertainties in the future of energy supply [2].

Agriculture is both cause and victim of water scarcity, as the largest consumer or water (70% of world demand) [3]. To minimize the contribution of agriculture to scarcity problems, reuse practices are a promising alternative, integrated into international environmental strategies; European and National Councils. However, agricultural reuse practices using poor water quality can result in health and environmental issues [4], since conventional urban wastewater treatment plants (UWWTP) are known to be the main pathways for contaminants entering the environment (including farming areas) through the water cycle [5]. Of these urban contaminants, Contaminants of Emerging Concern (CECs), mainly Pharmaceuticals, are of greatest concern [6–9]. So much so that some of them are regarded as priority substances in the area of water policy, in accordance with European directives such as the recently published European watch list of substances [Commission Implementing Decision (EU) 2022/1307 of 22 July 2022] [10, 11]. However, this concern could increase further in recent years, as a result of new consumption habits or the development of new pharmaceutical formulations. One example is the widespread use of pharmaceuticals during the COVID-19, increasing the release of pharmacological residues in aqueous matrices [12]. In this sense, an increase in the presence of antiviral drugs in urban wastewater of more than 70% has been reported compared to before and during the pandemic [13, 14]. Therefore, appropriate agricultural reuse policies are necessary.

New rules will apply from 26 June 2023 in all EU Member States for stimulate and facilitate water reuse thought the new (EU) 2020/741 on minimum requirements for water reuse [15] as part of an European Circular Economy Action Plan [16]. This new unifying legislative tool is the main driver to stimulate public acceptance of the free flow of agricultural crops irrigated with reclaimed water, especially after the absence of a common European Union environmental/health standard in previous years.

To update the UWWTP to meet the minimum requirements of (EU) 2020/741, two different approaches can be adopted: (1) implementation of new water treatment technologies that are effective, ecological, economical, and, if possible, easy to manage and cheap; or (2) optimize existing ones to meet those minimum requirements. In both cases, tertiary treatments which comply with microbiological thresholds are required [17], while no discharge limits are described for CECs. However, they are expected to be included in the coming years. Therefore, the design, establishment, and implementation of advanced tertiary treatments capable of increasing the water quality of effluents for agricultural use, ensuring compliance with the new EU requirements, and also preventing the emergence of future restrictive regulations are necessary.

Advanced oxidation processes (AOPs) have been recognized as a promising alternative for CECs removal [18–20], with solar TiO_2_-Heterogeneous photocatalysis being one of the most efficient and environmentally friendly technique [21–23], mainly due to the properties of TiO_2_ as its strong photocatalytic activity by generating unselective oxidizing agents, such as hydroxyl radicals (^·^OH) [24, 25]. However, there are still limitations to applying this type of tertiary treatment on a large scale mainly due day–night irradiation cycle, which limits the possibility of working continuously. Although efforts have been made to develop new photocatalysts capable of working in light and dark conditions [named “all-day-active photocatalyst” or “round-the-clock photocatalyst” (RTCPt)], the research in this field is still in its initial phase, and it may take decades to get an efficient catalyst at relatively low cost [26, 27]. Therefore, actually, a viable large-scale alternative is the combination of solar photoreactors with artificial UV-light sources.

Several studies have reported promising results about the performance of heterogeneous photocatalysis based on conventional UV-light lamps (i.e., mercury or xenon) [28–31]. However, they are relatively expensive and causes the generation of highly toxic waste [32]. Light-emitting diodes (LEDs), emerged in last decade, turning out as an alternative cost-effective competitive source of light due to their advantageous size, lifetime, wavelength and light distribution control, as well as environmental safety [33–35]. Therefore, considering the low availability of natural solar energy in some countries, the unavoidable time needed to study and test innovative photocatalysts and the increasing pressure on the availability of quality unconventional water resources, the combination in AOPs of solar radiation with alternative UV-LED sources is promising.

In addition, apart from the radiation source, other factors, such as the specific structure of the reactor, the radiation source position, the catalyst dosage, or the required final filtration step for catalyst removal, play an important role on the overall CECs’ removal and disinfection efficiencies [36, 37]. In spite of all this, real-scale studies covering all these issues as a whole are limited, and even more limited studies that include other factors such as the problems of system fouling, the periodicity of cleanings, and possibilities for improvement along with associated costs.

Under all of these perspectives, the goal of this work is the integration of a full-scale TiO_2_-heterogeneous tertiary treatment in a conventional UWWTP, working in continuous mode under artificial (UV-LED) and natural solar irradiation, to guarantee high-quality effluent line with reuse purposes in line with the current and future regulation’s requirement. Optimization of the system by evaluating different experimental parameters (optimal catalyst dose, type and/or arrangement of the UV radiation source) on the degradation efficiency of CECs and disinfection, together with the optimisation of the final filtration stage for catalyst recovery, as well as the costs involved, will help to obtain an overall view of the use of the system on a large scale.

## Materials and methods

### Chemical and reagents

A total of 12 pharmaceutical compounds from different therapeutic groups were selected: (1) one analgesic: Acetaminophen (ACT); (2) four antibacterial: Amoxicillin (AMX), Erythromycin (ERY), Tetracycline (TCL), and Sulfamethoxazole (SMX); (3) one anticonvulsant: Carbamazepine (CBZ); (4) one antimalarial: Chloroquine (CHL); (5) three anti-inflammatory: Diclofenac (DCF), Ketoprofen (KTP) and Naproxen (NPX); (6) one antipsychotic: Haloperidol (HLP); and, (7) one antidepressant: Trazodone (TRZ). The selection of the compounds was based on two fundamental aspects: (1) persistent or bio-recalcitrant compounds commonly found in effluents from Murcia Region UWWTP, location where the proposed tertiary system was installed [38, 39]; (2) compounds likely to appear in real effluents due to their use in new therapeutic strategies related to respiratory infection by SARS-CoV-2 according to Spanish Agency for Medicines and Medical Devices web pages [40, 41]. Analytical standards of selected pharmaceuticals (purity higher than 99%) were purchased from Sigma-Aldrich^®^. Physico-chemical properties of the selected compounds are described in Table S1.

For analytical measurements, MilliQ-water, methanol, and formic acid (all UHPLC-grade, ≥ 99.9%) were provided by Sigma-Aldrich^®^. Commercial AEROXIDE^®^ TiO_2_ P-25 (Evonik Industries, CAS. 13463-67-7) with a specific surface area of 35–65 m^2^/g and purity ≥ 99.5% was used as a catalyst.

### Analytical measurements

Different analytical and microbiological techniques were used to optimize the tertiary treatment and to determine effluent water quality after the photocatalytic process.

Total suspended solids (TSS), electrical conductivity (EC), pH, turbidity, chemical oxygen demand (COD), 5-day biological demand (BOD_5_), total and soluble phosphorus (Ptot, Psol), total nitrogen (TN), ammonium (NH_4_^+^), and nitrate (NO_3_^−^), analyses were carried out following Standard Methods (APHA, 2012) [42].

The pharmaceutical compounds were simultaneously monitored in CEBAS-CSIC analytical laboratories by liquid chromatography coupled with mass spectrometry using an UPLC–QTOF-MS system (Bruker Daltonik GmbH, Germany), equipped with an ACQUITY BEH C18 (100 mm × 2.1 mm, 1.7 µm) column described previously by Martinez-Alcala et al. [43]. For more information, see S1. Before injection in the chromatographic system, the samples were filtered using a 0.22 µm PTFE filter (Millipore). The detection limit (DL) for the selected compounds was adjusted to 5 µg/L with an associated error to each concentration level < 10%, using matrix calibration curves.

In addition, for the analysis of real samples containing pharmaceutical compounds below limits set, a sample pre-concentration step was performed by solid-phase extraction (SPE), allowing to decrease the detection limit by two orders of magnitude (detailed SPE procedure is described in S2). In all cases, target pharmaceuticals were quantified based on their peak area, by an internal standard approach using a linear regression. Results from SPE were adjusted using recovery tests (see Table S2).

*Escherichia coli* was analysed in fresh samples collected during experiments. Enumeration bacteria were performed by standard plate counting method using selective agar media (Chromocult^®^ (Merck)) according with ISO 9308-1:2014 [44]. For each analysis, 100 mL of water samples were filtered using a 0.45 µm-pore-size cellulose nitrate membrane (Sartorius) and obtained membranes were plated in petri dishes containing the selected medium. Later, plates were incubated for 24 h at 37 °C and counted. Detection limit was set at 1 CFU/100 mL (colony forming unit per mL), according to Class A maximum value (10 CFU of *E. coli*/100 mL) set by the new European Regulation on minimum requirements for water reuse ((EU) 2020/741).

Additionally, other microbiological indicators recommended for the control of Class A reclaimed water for agricultural irrigation according to [(EU) 2020/741] were analysed [15]. Under this perspective, pathogenic viruses (including total coliphages and somatic), and protozoa (*Clostridium perfringens* spores) were analysed thought an external ENAC certificated reference laboratory (IPROMA S.L) located in Castellón, Spain. For conditions, see S3.

### Tertiary treatment design

The experimental system was installed on February 2019 in the facilities of the Murcia Este UWWTP (managed by EMUASA), which is the largest treatment plant in Murcia region. Specifically, this UWWTP was stablished in 2000, with a total treatment capacity of 100,000 m^3^/day, designed for 833,000 population equivalents [45, 46]. The water line consists of a preliminary treatment and primary sedimentation, followed by an activated sludge process for the biological removal of nitrogen and phosphorus, using a specific configuration. The biological tank is divided in three identical lines (aerobic, anoxic, and aerobic), with a total volume of 41,405 m^3^ (6429 m^3^ anaerobic, 4850 m^3^ anoxic, and 30,146 m^3^ aerobic) and a final settling stage. This final stage has a feed stream connected directly to the proposed tertiary reactor.

**Fig. 1:**
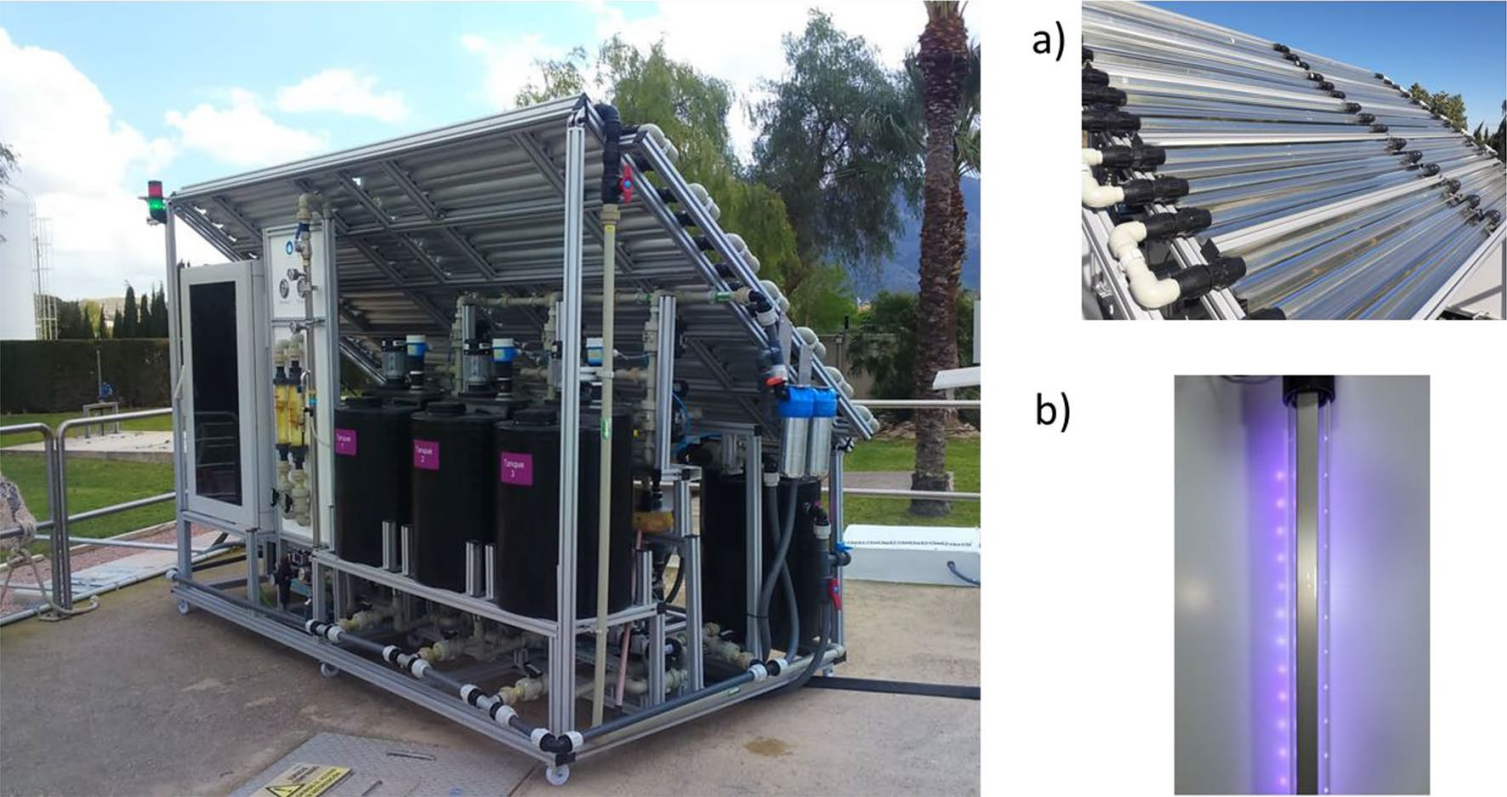
Tertiary treatment design, including the two photocatalytic working modes: **a** solar CPC photoreactor and **b** LED-UV lamps

The tertiary system (Fig. 1), with a maximum treatment capacity of 50 L/h, consists of three completely differentiated steps: (1) a filtration step (20 µm and 1 µm nylon series-mounted filters) to prevent blockages in the system; (2) the photocatalysis step; (3) a microfiltration final stage to recovery the catalyst (TiO_2_). It works in continuous mode due to its design configuration (3 tanks available). Thus, in the first tank (after filtration), the water is stored; in the second, the photocatalytic process is carried out; and the third is used for filtration. The hydraulic scheme of the proposed tertiary treatment is detailed in Fig. S1.

To work in the day–night radiation cycle, two photocatalytic systems are integrated: (1) based on solar Compound Parabolic Collector (CPC) and (2) based on artificial UV-LED lamps. The design allows to work by both technologies independently or combined.

Specifically, the CPC design consists of two CPC units containing a total of 24 borosilicate glass tubes (2.9 cm diameter) installed on a platform titled 37° from horizontal position. The total illuminated area was 4.25 m^2^, the total volume (TV) was 127 L, and the illuminated volume was 28.33 L (see Fig. 1a), while different configurations of LED lamps were tested (see 2.4). As the system is outdoors and is not thermally controlled, the temperature inside the reactor was continuously monitored by a control panel installed in the system to avoid overheating of UV-LED lamps.

Regardless of the photocatalytic process used, the catalyst was recovered by a final microfiltration step using commercial membranes installed in series producing two effluents, the permeate, and a mixture of catalyst/concentrated water which is returned to the input current. Selected membranes were based on ceramic materials mainly due to its high efficiency compared to polymer membranes, according to Jiménez et al. [47].

### Experimental procedure and sampling

The experimental procedure was designed according to a system optimization to achieve the highest technical and economic efficiency from different points of view: (1) catalyst dosage optimization; (2) efficiency of photocatalysis mode (natural sunlight, artificial light or a combination); (3) comparison of different UV-LED radiation source configurations; and (4) optimization of the efficiency of the final filtration stage testing different commercial membranes.

Under this perspective, and before to work with real wastewater effluents in continuous operation mode, different experimental sets were conducted using real effluents fortified with 200 µg/L of selected pharmaceuticals. For that, required volumes of stock solution containing pharmaceutical compounds were directly added to real wastewater into reaction tank. This initial concentration was chosen, because it is a sufficiently high concentration to obtain degradation values using available analytical techniques, and a low enough concentration to simulate real environmental conditions, due pharmaceuticals are found in real wastewaters in the ng/µg range [48]. To assess a completely realistic scenario, experiments were performed at natural secondary effluent pH (7.44 ± 0.21). All samples were collected in 1-L amber glass bottles and taken directly to the laboratory for further analysis. For SPE analysis, samples were stored at 4 °C and extracted within 1 day of collection.

In the experimental line, three catalyst concentrations (0.1, 0.5, and 0.75 g/L) in the three photocatalytic possible configurations [solar photocatalysis using CPC (SOLAR mode), artificial photocatalysis using UV-LED (UV-LED mode), and the combination of both (SOLAR + UV-LED)] were tested. Additionally, experiments were also performed without TiO_2_ (photolytic mode). Although the total treated volume for combined mode can reach 127 L, to compare the removal efficiency of CECs under the three photocatalytic modes, the initial experiments were performed using 85 L, which is the maximum volume to be used in experiments using only UV-LED mode. Solar photocatalytic fortified experiments were done in the morning on completely sunny days with an average radiation of 35 W/m^2^. Also, two configurations in UV-LED source design were tested (Table 1):

(i) An initial commercial design containing two annular photoreactors provided by adjustable UV-A power (Photobench LED365-32, 40 LED/UV-A lamps per device), and refrigeration through forced air convection (Strip mode). The total irradiated volume was 1.77 L.
(ii) A self-designed design according with the needs of plant included within the mixing tank itself (Bar Mode). In particular, the total irradiated volume was increased (from 1.77 L to 85 L) by the construction of 2 UV-A lamps with an intensity of 54 W each. For the design, an aluminium base bracket (20 × 10 × 1.3 mm) was used. In this configuration, an additional ventilation system was installed, to avoid overheating and breakage of the lamps, using a series of electronic components and fans.

**Table 1:** UV-LED source and design

UV-LED type	*λ* (nm)	Configuration	Power (W/m^2^)
Strip	365	In specific module	35, 130, 240
Bar	365–425	Inside the reactor	54, 108,

For final filtration step, four tubular microfiltration membranes (M1–M4), based on α-aluminium oxide (Al_2_O_3_), with different membrane surface areas (0.24, 0.5, 0.38, and 0.43 m^2^, respectively) were tested. M1–M3 were provided by Likuid (San Sebastián Gipuzkoa, Spain), while M4 was provided by Atech innovations GmbH (Gladbeck, Germany). Detailed information about the physico-chemical properties of each of them is shown in Table S3.

## Results and discussion

### Inlet wastewater monitoring

The input current to the system was monitored approximately once a month during all experimental period (from February 2019 to February 2022) to assess the variability in real wastewaters. Analytical results regarding physico-chemical, microbiological, and pharmaceutical content are detailed in Table 2.

**Table 2:** Characterization of inlet wastewater stream to the photocatalytic system (*n* = 20)

Physico-chemical parameters		Pharmaceuticals		Microbiological indicators	
pH	7.44 ± 0.21	ACT (µg/L)	0.20 ± 0.20	*E. coli* (CFU/100 mL)	1.00 × 10^4^ ± 1.00 × 10^4^
EC (mS/cm)	2019.29 ± 325.61	AMX (µg/L)	n.d	Total coliphages (PFU/100 mL)	178.00 ± 139.0
Turbidity (NTU)	11,27 ± 5,68	CBZ (µg/L)	0.10 ± 0.11	Somatic coliphages (PFU/100 mL)	176.00 ± 140
TSS (mg/L)	36.21 ± 17.60	CHL (µg/L)	n.d	*Clostridium perfringens* spores (CFU/100 mL)	783.00 ± 714.00
COD (mg/L)	36.28 ± 35.00	DCF (µg/L)	0.60 ± 0.08		
TN (mg/L)	53.69 ± 36.96	ERY (µg/L)	0.10 ± 0.14		
DBO_5_ (mg/L)	4.14 ± 1.77	HLP (µg/L)	n.d		
NH4^+^ (mg/L)	3.59 ± 2.78	KTP (µg/L)	0.54 ± 0.22		
NO_3_^−^ (mg/L)	5.26 ± 15.42	NPX (µg/L)	0.46 ± 0.10		
P_tot_ (mg/L)	2.88 ± 2.15	SMX (µg/L)	0.48 ± 0.24		
P_sol_ (mg/L)	2.95 ± 2.43	TCL (µg/L)	n.d		
		TRZ (µg/L)	n.d		

*n.d.* non-detected, *PFU* plaque-forming unit

On the physico-chemical parameters analysed, the pH remained constant with a value of 7.44 ± 0.21. However, greater deviations were found for the rest of the parameters, demonstrating the variability when working with real wastewater. In view of this variability, and considering that TSS and high turbidity values may affect the optical capacities of the water to be treated and therefore the photocatalytic performance [49], a pre-filtration step was included in the system by incorporating two nylon filters arranged in series (20 µm and 1 µm). This initial improvement resulted in a decrease in turbidity and TSS values to 2.85 ± 1.86 and 6.33 ± 4.31, respectively. In addition, an average decrease in COD of 22% was detected. This led to a decrease in the number of chemical cleanings required to ensure the efficiency of the process, going from a monthly chemical cleaning to a bi-monthly cleaning, thus reducing in a 50% the costs related to the acquisition of cleaning reagents.

On the other hand, of the 12 pharmaceuticals selected, only 7 were found in the water entering the system. Despite the type and concentration of pharmaceuticals varied from day to day, values were always in the µg/L range. After the low concentrations found, it was decided to fortify the water entering the system with a known concentration of pharmaceuticals (200 µg/L), with the aim not only of optimizing the system, but also considering extreme situations, where inlet wastewater may contain other types of contaminants of emerging concern or high concentrations of them caused by point discharges.

### Influence of TiO_2_ dose and operational mode in pharmaceuticals removal

**Fig. 2:**
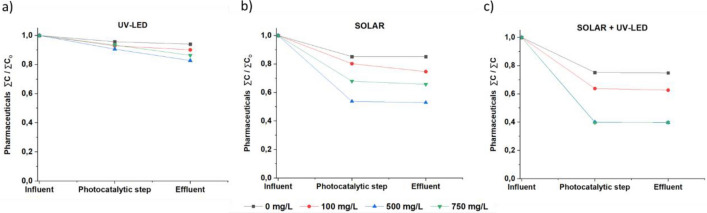
Catalyst dosage optimization (pH 7.44, irradiation time = 30 min)

The effect of TiO_2_ dosage (0, 0.1, 0.5, and 0.75 g/L) on pharmaceuticals removal under the three operational modes: UV-LED, SOLAR, and SOLAR + UV-LED mode was evaluated using the initial commercial configuration [LED/UV-A design and M3 membrane (see 3.5)] adapted with the pre-filtration step in order to minimize the inlet water variability. To obtain comparable results for all different working modes, all experiments were performed with an average radiation of 35 W/m^2^ and using 85 L as a total wastewater volume. The removal pharmaceutical rates (from 200 µg/L fortified wastewaters) compounds after 30 min of irradiation time are presented graphically in Fig. 2.

The results revealed that the photocatalysis oxidation activity is improved with increasing the TiO_2_ dosage, regardless of the operational mode. i.e., increasing the dosage from 0 (photolytic mode) to 0.5 g/L, improved the removal efficiency in a 5%, 32%, and 36% when UV-LED, SOLAR, and SOLAR + UV-LED photocatalytic modes were performed. This improvement can be attributed to the growth of active sites through TiO_2_, which acts as a semiconductor in the photocatalysis process. Consequently, the formation of reactive hydroxyl radicals and electron–hole pairs on the surface of TiO_2_ material increases, improving pharmaceutical oxidation reactions [50]. However, when the catalyst dose was increased from 0.5 to 0.75 g/L, a decrease in the pharmaceutical removal efficiency along with an increase in turbidity (> 300%) was observed in UV-LED and SOLAR modes, while for combined mode, the pharmaceutical removal percentage remained constant. This is mainly due to the fact that at high doses of catalyst, particle agglomeration can occur, decreasing the active sites on the TiO_2_ surface, affecting photocatalytic and photolytic degradation mechanisms and, therefore, decreasing the removal efficiency of pharmaceutical products [51–53].

Several authors have shown that the catalyst dose is a determining aspect in persistent pharmaceuticals degradation efficiency, both using solar radiation and UV-LED lights. Biancullo et al. [54] reported a degradation rate improvement by increasing the catalyst dosage from 0.1 to 1 g/L using UVA-LEDs, while with higher TiO_2_ concentrations, the degradation was hindered. Al-Furaiji et al. [55] studied the same catalyst concentration range in a CPC photoreactor using natural sunlight. The results confirmed 0.6 g/L as the optimal dose of catalyst, while higher doses did not improve the process. A similar pattern was observed by Jalloui et al. [56] using different UV-LED lamps’ configurations, who also reported similar degradation efficiencies when 1.0 g/L and 1.5 g/L TiO_2_ dosages were used in the photocatalytic process.

However, and although most of authors propose an optimal catalyst range between 0.5 and 5 g/L, the optimal catalyst dosage depends primarily on the reactor design, and the volume and characteristics of the water to be treated [57, 58]. Therefore, prior catalyst dose optimisation is necessary when designing a large-scale system [34].

On the other hand, UV-LED mode showed low pharmaceuticals disposal values (9.4%) compared to solar mode (46.3%), while combined mode showed the highest efficiencies (61.3%). Because the radiation was similar in all cases, these differences can be attributed to the total irradiated volume. In the initial commercial configuration of the system, the total irradiated volume in UV-LED mode was only 1.77 L, compared to the volume irradiated using SOLAR mode (28.33 L), whereas when SOLAR + UV-LED combined mode is used, the irradiated volume is the sum of the two above. Furthermore, in all cases, the addition of TiO_2_ produced significantly higher rates of pharmaceutical degradation compared to UV photolysis [59]. No significant differences were observed comparing removal efficiencies before and after the photocatalytic process (after the final filtration stage) in SOLAR and SOLAR + UV-LED modes, confirming that the commercial filtration membranes used have no effect on pharmaceuticals removal, while a slight decrease (maximum value of 7.9%) was found in UV-LED mode, attributed to possible membrane damage during experiments.

**Fig. 3:**
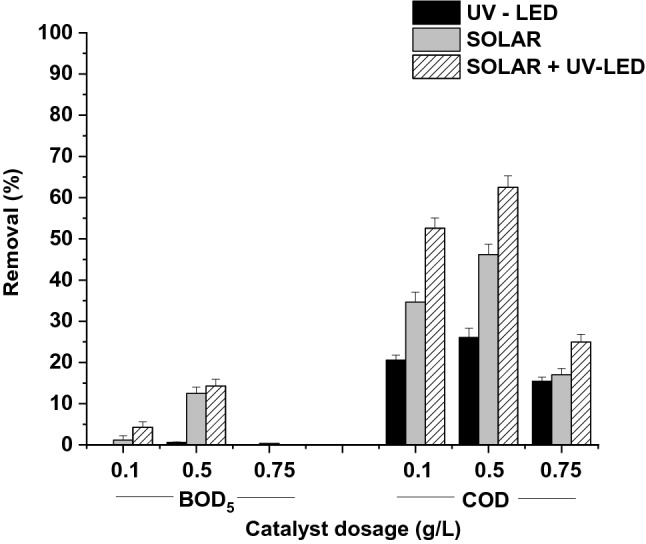
BOD_5_ and COD removal efficiencies under the different catalyst dosages (0.1, 0.5, and 0.75 g/L) and photocatalytic modes (UV-LED, SOLAR, and SOLAR + UV-LED)

Same trends were observed for mineralization in the photocatalytic system. DOC and BOD_5_ removal increased with the catalyst dosage, and higher values were obtained for 0.5 g/L, achieving removal percentages of 62.5 ± 2.8% and 14.3 ± 1.6%, respectively, when SOLAR + UV-LED mode was performed (Fig. 3). In addition, the removal efficiency followed the trend: UV-LED < SOLAR < SOLAR + UV-LED, demonstrating once again a relationship between the irradiated volume and the decrease in organic matter.

### Effects of irradiation time

**Fig. 4:**
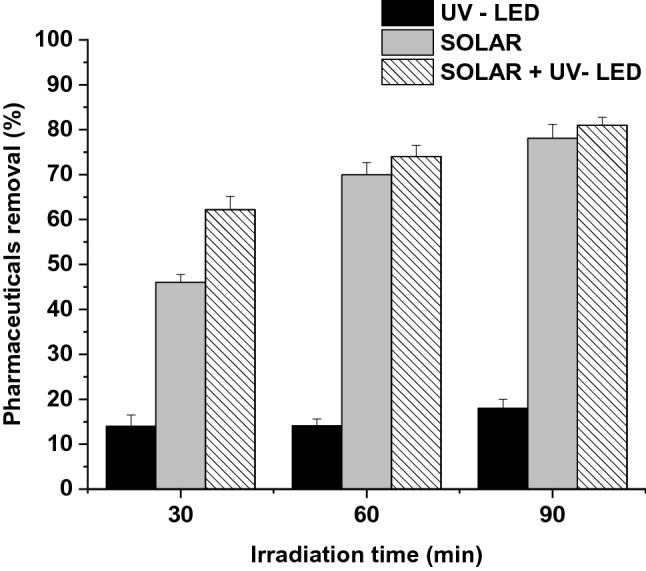
Effects of irradiation time in pharmaceuticals removal in the three photocatalytic modes (UV-LED, SOLAR, and SOLAR + UV-LED)

The radiation time required for the photocatalytic process under the selected optimal catalyst dose (0.5 g/L) as a function of the different photocatalytic modes was considered. Results of the pharmaceuticals removal under three different irradiation times (30, 60, and 90 min) are reported in Fig. 4.

As expected, a gradual increase in removal efficiency through irradiation time exposure was observed all cases, with values ranging from 14.0 ± 2.5 to 18.0 ± 2.2, 46.0 ± 1.8 to 78.1 ± 3.1, and 62.2 ± 3.0 to 81.0 ± 1.8 when UV-LED, SOLAR, and SOLAR + UV-LED modes were used, respectively. Lower efficiencies were obtained for UV-LED mode, as reported in Sect. 3.2, demonstrating that, despite the increase in contact time, the irradiated volume would not be sufficient for pharmaceuticals removal. On the other hand, no large differences were observed in pharmaceuticals removal between 60 and 90 min, mainly due to compounds with high persistence, suggesting the need for longer reaction times, or the addition of agents capable of accelerating the reaction such as H_2_O_2_ form complete mineralization [60]. Specifically, ERY and CBZ were the pharmaceuticals with the lowest degradation rates, showing maximum removal percentages of 45% and 61%, respectively, in combined SOLAR + UV-LED mode. This can be attributed to the neutral character of both compounds, which hinders their protonation at neutral pH, decreasing their reactivity, so, to obtain higher efficiencies, it would be necessary to work at lower pH values (when the pH is much lower than p*K*a) [61, 62].

Carabin et al. [63] demonstrated the influence of pH in carbamazepine removal photocatalytic reaction, showing removal percentages of 60% at pH 7, while the higher removal efficiencies were obtained working at pH 5 (80%). Similar results were reported by Li et al. [64], increasing the ERY removal efficiency from 30 to 50% when pH dropped from 7 to 5. A similar behaviour was observed for acetaminophen. In this case, SOLAR and SOLAR + UV-LED removal efficiencies at 30 min of irradiation time showed percentages of 52% and 62%, respectively. However, in this case, the increase in exposure time resulted in higher removal efficiencies (around 97% in both cases). This is mainly because, despite being a neutral compound, with a p*K*a similar to ERY, it has a lower molecular weight. Similar results were reported by Zhang et al. [65]. The authors reported a 20% of removal using 30 min of irradiation time and working at neutral pH with a catalyst dose of 0.5 g/L (optimal value according to 3.2), while increasing the time to 100 min, 50% of removal percentage was achieved.

However, and although the pharmaceutical properties have a significant influence on the photocatalytic reaction efficiency, for the implementation of a large-scale system, it is necessary to assess the degradation percentages as a whole, since other factors such as the presence of other contaminants, ions, or the organic matter present in real waters is a key factor in photocatalytic reaction [66–68].

### UV-LED source efficiency

**Fig. 5:**
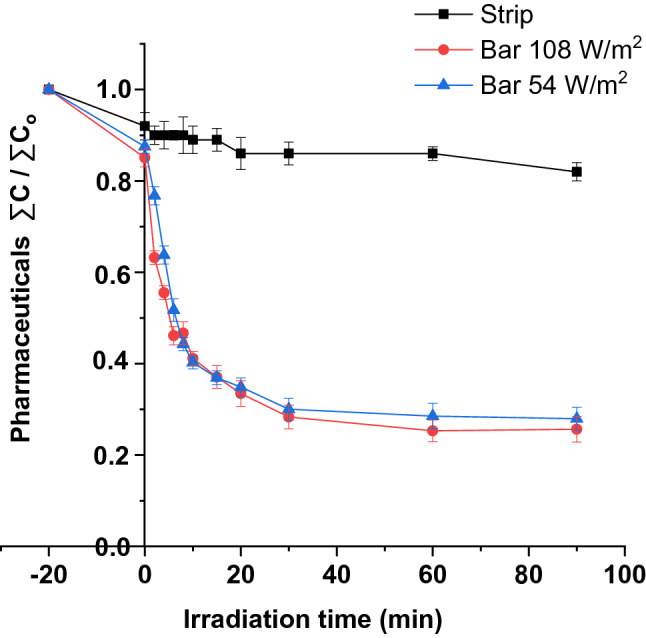
Pharmaceuticals removal under different UV-LED configurations

Following the results obtained in paragraph 3.2 and 3.3, and with the aim of improving the UV-LED efficiency, a study was carried out increasing the power of the lamps from 35 to 239 W/m^2^, which corresponds to the maximum power that the lamps can support. The results showed only a 3% increase in pharmaceutical removal efficiency, while energy consumption increased by 0.23 kW/h (from 2.19 to 2.33 kW/h). According to these poor results, and since the photon absorption rate is directly related to the type of photoreactor and the construction materials [69], it was decided to remove the commercial module where UV lamps were included by inserting a self-designed 54 W/m^2^ UV-LED lamp (bar mode) into the photocatalytic tank (form detailed configuration, see Fig. S1). Results of the new configuration showed higher removal efficiencies (74.4 ± 2.8%) compared with the maximum strip mode removal percentage values (18 ± 2.2%), see Fig. 5.

This increase was mainly due to the fact that, in this new configuration, the whole volume of water is in contact with the UV-LED lights during the reaction time, thus increasing the irradiated volume from 1.77 to 85 L. According to these results, and to further improve efficiency, a second self-designed lamp was introduced into the photocatalytic reactor, thus obtaining total radiation of 108 W/m^2^ into the tank. However, despite expectations, similar degradation curves were obtained in both experiments. This may be associated with the recombination of photo-produced electron–hole pairs at high light intensity, resulting in decreased photocatalytic efficiency, because the mass transfer limit has been reached [70].

In addition, in both cases, the degradation efficiency was increased by 30 min, while from this irradiation time, it remained constant. About dark absorption tests, initially applied to establish the affinity between selected pharmaceuticals and the catalyst under different UV-LED configurations, did not show significant differences in all cases.

After the results obtained, and with the aim of working during the night, or during non-sunny days, the system was adjusted to a single self-designed lamp (54 W/m^2^) continuous work mode.

### Microfiltration final step

The commissioning of the system presented several operational problems when commercial M1 (0.24 m^2^ surface area) and M2 (0.5 m^2^ surface area) membranes were used in the final filtration step for catalyst removal. The main limitation was observed in the permeate flow rate, resulting in the shutdown of the reaction and filtration tanks due to the high-water level in the system associated with the blocking of membrane pores by the TiO_2_ particles [71]. This incidence was corrected by reducing the water flux in the system (initially designed to treat 50 L/h) by 30%. However, the need for daily chemical (acid/base) cleanings limited the system’s ability to work in continuous mode. Therefore, to reach the required permeate workflow and avoid membrane fouling problems, the system configuration was modified, increasing the contact surface using two commercial membranes arranged in series. In this new configuration, M3 (0.76 m^2^ of total surface area, 0.38 each) and M4 (0.86 m^2^ of total surface area, 0.43 each) membranes were tested. Total removal percentages of TSS and *E. coli* were obtaining for both membrane types working in 60 min of irradiation time. However, greater differences were observed for other physico-chemical parameters. Specifically, removal percentages of COD and turbidity during microfiltration step showed percentages of 29% and 41%, respectively, for M3, while higher removal efficiencies were obtained for M4 (43% and 51%) (more information in Fig. S2). In addition, fouling problems were greater when M3 was used, with residue crystallization observed inside the membrane. This resulted in the need for a monthly chemical cleaning (acid/base) and a membrane replacement every 3 months of operation. However, in the case of M4, an optimal membrane operating time of 6 months (3 months more than for M3) was established, while a monthly chemical cleaning periodicity was also necessary with the aim of extending the membrane’s life time, according with Duraisamy et al. [72].

### Agronomic quality of system effluents

The most recent EU directive on “Minimum requirements applicable to reclaimed water for agricultural irrigation” (EU, 2020/741) [15] was selected as a reference to establish whether the treated water in the proposed and optimized Full-Scale photocatalytic system was suitable for agricultural use. In particular, this directive, which will be directly applicable in all Member States from 26 June 2023, sets four different "classes" based on the irrigation water quality (A, B, C, and D), A being the most restrictive class. Under this perspective, a detailed characterization of the effluents generated in the system working in continuous mode and under different photocatalytic modes was carried out.

The results showed that, regardless of the photocatalytic mode, and the irradiation time, the values for DBO_5_, TSS, and Turbidity were below the most restrictive values included in the EU regulation (class A) (BOD_5_ ≤ 10 mg/L, TSS ≤ 10 mg/L, and Turbidity ≤ 5 NTU).

**Fig. 6:**
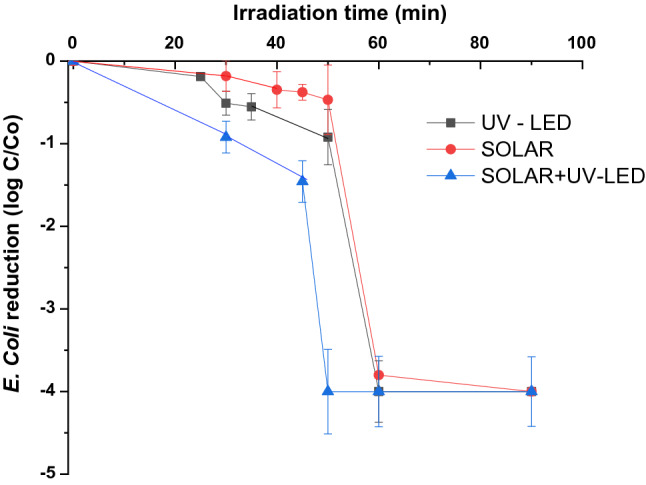
*E. coli* removal under the three photocatalytic modes: UV-LED, SOLAR, and SOLAR + UV-LED

On disinfection purposes, at 60 min of irradiation time, all system effluents showed a total reduction of *E. coli*, showing levels below the European regulation on minimum quality requirements (MQR) established for this bacterium (< 10 CFU/100 mL), the value marked for "the point of compliance" in “class A” reuse (Fig. 6). Shorter irradiation times, however, could be used when lower quality is required, so disinfection becomes a crucial stage in the proposed tertiary system. On the other hand, results showed that the combined mode (SOLAR + UV-LED) is the most effective for *E. coli* removal, so the continuous work in this mode could shorten the necessary irradiation times by more than 10 min/h, increasing the volume of daily treated water.

Additionally, and setting an exposure time of 60 min for all working modes, a monitoring of other microbiological indicators newly recommended for the control of “class A” reclaimed water was performed, including pathogenic and protozoa.

In particular, coliphages were considered as the best predictors of viruses, due to their high environmental persistence, and that their incidence and survival in aquatic environments are more similar to that of human enteric viruses than the traditional used bacterial indicators [73]. Results showed mean levels in inlet system current of 178 ± 139 PFU/100 mL for total coliphages and 176 ± 140 PFU/100 mL for somatic coliphages, lower results than reported by other authors in real wastewater samples [74, 75] probably due to the specific characteristics of viral contamination in the population. These values were drastically decreased in the output of the system, showing mean levels of 1. ± 0 PFU/100 mL for total coliphages, while somatic coliphages were non-detected.

On the other hand, *Clostridium perfringens spores* was selected as a protozoa indicator due to the ability of these microorganisms to be widely distributed in wastewater [17]. In addition, they are resistant to conventional tertiary treatments (chlorine, UV-C light, etc.), so more powerful treatments are required to reduce their levels compared to other parasites [76]. The follow-up of this parameter showed input levels to the system of 783 ± 714 CFU/100 mL, values similar to those published by Vivar et al. and Liu et al. [77, 78], while a decrease of almost 100 times was found in the output, with values of 10 ± 1 CFU/100 ml, results comparable to those obtained by other authors in advanced disinfection systems [79, 80].

According with this results, high agronomic quality regardless of the photocatalytic mode used was obtained, so continuous work using day-mode SOLAR and night-mode LED-UV could ensure the highest treatment capacity, up to a maximum treatment value of 438 m^3^/year, except for occasional stops due to chemical cleaning.

### Cost assessment

In relation to the technical–economic feasibility of this technology, Table 3 summarizes the economic cost associated with maintaining the photocatalytic system before and after optimization (excluding installation costs). These costs were estimated considering continuous mode system operation (using day-mode SOLAR and night-mode UV-LED) and a maximum treatment flow of 5000 m^3^/day, according with Prieto-Rodríguez et al. [83].

**Table 3:** Operation and maintenance costs of the system before and after optimization

	Initial configuration	After optimization
Routine maintenance and operation^a^	1.97 €/m^3^	0.78 €/m^3^
Chemical cleaning and consumables pre-treatment	2.10 €/m^3^	1.13 €/m^3^
Membranes	0.65 €/m^3^	0.65 €/m^3^
Catalyst	0.07 €/m^3^	0.04 €/m^3^
TOTAL €/m^3^ treated wastewater	4.79 €/m^3^	2.60 €/m^3^

^a^Cost time maintenance and operation: 15 €/h

A reduction of up to 45% of treated water costs was observed following improvements to the system (from 4.79 to 2.60 €/m^3^). The greatest differences were found for maintenance and operation items, showing a reduction of 60% compared to the initial configuration. This was mainly due to integration of the photocatalytic system into a digital platform, allowing a greater autonomy of the system, thus reducing personnel costs. Similar results were obtained for chemical cleaning and consumables pre-treatment items. In this case, a total reduction of 46% was achieved mainly due to the decrease in the number of cleanings of the system according two fundamental reasons: (1) working under optimal conditions (catalyst dose, adequate irradiation time, etc.) helped to reduce fouling problems; and (2) the introduction of nylon filters as a pre-treatment step protected the fouling of the same. In the latter case, although filtration consumable costs have been incorporated, the total cost was reduced by reducing cleaning reagent costs by up to 28%, due mainly to the fact that water has a lower concentration of organic matter due to the pre-filtration stage.

Despite the optimization of the final filtration phase, the costs associated with the acquisition of the membranes were not reduced. This was mainly due to the fact that the price of M4 was twice the price of M3, so that, from an economic point of view, it would not matter to work with one membrane or another. In general, it is reported that the main drawback of the use of membranes is the high cost of capital, along with the high energy required and the reagents for cleaning them, contributing significantly to an increase in costs [47]. This is why, specific membrane design studies are currently being carried out, with capacity and self-cleaning, allowing an improvement in both efficiency and costs [81]. However, further research is still needed.

Despite the cost reduction during optimization, estimated costs were relatively high compared to those reported in similar processes by other authors (total wastewater maintenance costs between 0.19 €/m^3^r and 0.36 €/m^3^) [82, 83]. This is mainly due to the fact that most of the studies carried out to date correspond to experiments in batch mode and using small-medium/scale prototypes, allowing to treat volumes that are only around 500 m^3^/year. In addition, the costs of plant operators and reagents required for cleaning, which are necessary to work continuously, are often not considered. However, when working with such a system on a large scale, proper and continuous cleaning of the system is necessary, avoiding not only soiling, but extending the life of the system, and also avoiding problems of microbial proliferation in the ducts, since, inadequate cleaning could also lead to algal blooms, and therefore to reduced efficiency. This means that, for large-scale work with such systems, the cost of maintenance and operation could be ten times higher. Therefore, further optimization and system design is necessary to be able to implement these systems on a large scale. On the other hand, the coupling of photocatalytic systems with other more economical technologies could improve yields, reducing costs.

## Conclusions and future perspectives

An integral full-scale photocatalytic system, capable of working during the day-night irradiation cycle, was installed in a conventional UWWTP with agricultural reuse purposes. System optimization studies showed the highest pharmaceutical removal efficiencies using 0.5 g/L of TiO_2_ and combined SOLAR + UV-LED photocatalytic modes. Self-designed lamp (bar) as UV-LED radiation source showed higher CECs removal efficiencies than commercial lamps, while longer surface area membranes decreased fouling problems and cleaning frequency.

The effluents of the system met the criteria established by the new European legislation on reuse (EU 2020/741), thus guaranteeing high-quality agronomic effluents in all photocatalytic modes. However, 60 min of irradiation time would be needed to reach “class A” quality, while shorter times could be used in less restrictive qualities.

After the optimisation stage, total costs were reduced by 45%. However, economic and energy ratios need to be further improved with a view to their large-scale implementation, with the aim of obtaining high-quality treated water at competitive costs.

Obtained results will be useful in the near future, where regulations will be more restrictive, including limits for pharmaceuticals. Furthermore, this real case study opens the way to future lines of research focused on the application of UV-LED lamps on advanced treatments in areas with little or no solar radiation. Knowledge of the most influential operating parameters and devices available on the market can also help improve large-scale photocatalytic efficiency, since a well-designed reactor can reduce waste or energy and catalyst, improving the associated costs. All these aspects are key to the implementation of new climate change initiatives, contributing to the achievement of sustainable development goals, promoting water savings.

## Supplementary Information

Below is the link to the electronic supplementary material.

Supplementary file1 (DOCX 478 kb)
