# Supplementary material for: Integrated full-scale solar CPC/UV-LED–filtration system as a tertiary treatment in a conventional WWTP for agricultural reuse purposes
Source: Photochem Photobiol Sci. 2022 Nov 19;22(3):641–54. doi: 10.1007/s43630-022-00342-9 (PMC9676787; doi:10.1007/s43630-022-00342-9)
Supplement: Supplementary file 1 — Supplementary file1 (DOCX 478 kb) [file 43630_2022_342_MOESM1_ESM.docx]

**SUPPLEMENTARY INFORMATION**

1. **General information**

**S1. UPLC-QqTOF-MS analysis for water samples**

A liquid chromatography-quadrupole time-of-flight mass spectrometry (UPLC-QqTOF-MS) system was used in order to identify and quantify the pharmaceutical compounds selected in this study. Specifically, LC separation was achieved using an ACQUITY I-Class UPLC system (Waters Corporation, Milford, MA, USA) coupled with an ACQUITY BEH C18 (100 mm x 2.1 mm, 1.7 µm) column. Mobile phases were 0.1% formic acid in MilliQ water (solvent A) and 0.1 % MeOH (solvent B). The gradient used ranged from 5% to 95% of solvent B: Initially was increased from 5% to 25% in 1.50 min, after increased from 25% to 80%, kept at 80% during 1 min, increased in 8 min and from 80% to 95% in 1 min and finally returned to its initial conditions in 4 min. The total analysis run time was 15.50 min. The flow rate was adjusted at 0.25 mL/min and 10 µL of sample was injected. The LC system was connected to a Bruker Daltonics, maXis q-TOF mass spectrometer equipped with an electrospray ion source (Bruker Daltonics, Bremen, Germany). The ion source settings were as follows: nitrogen was used as nebulizer, drying and collision gas; the nebulizer pressure was 2 bars, the drying gas flow was 8 L/min, the dry gas temperature was 200 ºC and the capillary voltage was 4500 V. The system worked via TOF MS survey scan (resolving power ≥ 55000 FWHM). The target pharmaceuticals were identified and reported from accurate-mass scan data using the software Target Analysis (1.3) and Data Analysis (4.2) from Bruker. In order to simplify, all analysis was performed in positive ionization mode.

**S2. Solid-phase extraction (SPE) protocol**

SPE was performed with commercial Oasis® HLB (200 mg, 6 cm^3^) using a Visiprep vacuum manifold. Before use, the cartridges were conditioned with 4 ml of methanol and 4 ml of Milli-Q water (pH adjusted to 8, same pH as the real water samples, in order not to alter their composition). Samples (100 ml) were passed through the cartridges at a flow rate of 1 ml/min. The cartridges were then rinsed with 4 ml of ultrapure water and vacuum dried for 10 min to remove excess of water. Finally, the retained compounds were eluted with 8 ml MeOH (4 + 4). The extracts were evaporated to dryness under a gentle nitrogen stream and reconstituted with 1 ml of H_2_O/MeOH (95:5, *v/v)* containing 0.1% formic acid. Final extracts were filtered through a 0.22 μm PTFE filter.

**S3. Pathogenic viruses and protozoa analytical procedure**

Levels of somatic coliphages were quantified by the double-layer agar (DAL) plaque assay technique using as the host strain E. coli C (ATCC 13706) and E. coli WG5 (ATCC 700078). One or five millilitres per concentrate per samples were treated with (10%) of chloroform to disrupt all the bacteria cells. All samples were centrifuged at 2500 ×g for 5 min at 4 °C and the supernatant was filtered. For the lower and upper layers in the double agar test, tryptone-yeast- extract glucose (TYG) agar and TYG semisolid agar. Plates were incubated at 37 °C for 24 h and the levels of total coliphages were expressed in plaque forming units per 100 mL (PFU/100 mL) as described ISO 10705-2. In addition, DAL plaque assay was used to quantify of total coliphages was applied after the concentration of 100 mL water samples (Méndez et al., 2004), as recommended ISO 10705-3.

For the analysis of Clostridium perfringens spores, one millilitre of this thioglycollate culture was inoculated in sporulation broth and incubated at 37 C for 72 h in anaerobic conditions. After the incubation, the spores were recovered by centrifugation (10,000 rpm, 15 min). The cultures were washed and the suspension of spores was prepares as described ISO 14189.

1. **Tables**

**Table S1**. Physico-chemical properties of selected pharmaceutical compounds

|  | **Drug type** | **Formula** | **Molecular Weight**  **(g/mol)** | **Log Kow** | **pKa** |
| --- | --- | --- | --- | --- | --- |
| ***Acetaminophen***  ***(ACT)*** | Analgesic | C_37_H_67_NO_13_ | 151.16 | 3.06 | 8.9 |
| ***Amoxicillin***  ***(AMX)*** | Antibacterial | C_16_H_19_N_3_O_5_S | 365.4 | 0.87 | 2.6 |
| ***Carbamazepine***  ***(CBZ)*** | Anticonvulsant | C_15_H_12_N_2_O | 236.3 | 2.45 | 15.9, -3.8 |
| ***Chloroquine***  ***(CHL)*** | Antimalarial | C_18_H_26_ClN_3_ | 319.9 | 4.63 | 10.1 |
| ***Diclofenac***  ***(DCF)*** | Anti-inflammatory | C_14_H_11_C_l2_NO_2_ | 296.1 | 4.51 | 3.9 |
| ***Erythromycin***  ***(ERY)*** | Antibacterial | C_37_H_67_NO_13_ | 733.9 | 3.06 | 8.8 |
| ***Haloperidol***  ***(HLP)*** | Antipsychotic | C_21_H_23_ClFNO_2_ | 375.9 | 4.30 | 8.65 |
| ***Ketoprofen***  ***(KTP)*** | Anti-inflammatory | C_16_H_14_O_3_ | 254.3 | - | 3.98 |
| ***Naproxen***  ***(NPX)*** | Anti-inflammatory | C_14_H_14_O_3_ | 230.26 | 3.18 | 4.18 |
| ***Sulfamethoxazole***  ***(SMX)*** | Anticonvulsant | C_10_H_11_N_3_O_3_S | 253.28 | 0.89 | pKa1 = 1.6 pKa2 = 5.7 |
| ***Tetracycline***  ***(TCL)*** | Antibacterial | C_22_H_24_N_2_O_8_ | 444.4 | -1.37 | 7.68, 3.3 |
| ***Trazodone***  ***(TRZ)*** | Antidepressant | C_19_H_22_ClN_5_O | 371.9 | 3.21 | 6.79 |

**Data from PubChem (*[*https://pubchem.ncbi.nlm.nih.gov/*](https://pubchem.ncbi.nlm.nih.gov/)*) and Network of reference laboratories, research centres and related organisations for monitoring of emerging environmental substances (*[*https://www.norman-network.net/*](https://www.norman-network.net/)*)*

**Table S2**. SPE recovery test for selected pharmaceuticals.

| **Compound** | **Recovery (%)** |
| --- | --- |
| ***ACT*** | 88 ± 4 |
| ***AMX*** | 71 ± 8 |
| ***CBZ*** | 97 ± 2 |
| ***CHL*** | 82 ± 5 |
| ***DCF*** | 78 ± 6 |
| ***ERY*** | 84 ± 7 |
| ***HLP*** | 81 ± 6 |
| ***KTP*** | 95 ± 3 |
| ***NPX*** | 92 ± 4 |
| ***SMX*** | 91 ± 5 |
| ***TCL*** | 85 ± 7 |
| ***TRZ*** | 79 ± 6 |

**Values based on triplicate spiked samples analysis*

**Table S3**. Properties of commercial ceramic microfiltration membranes tested.

|  | **Membrane types** | | | |
| --- | --- | --- | --- | --- |
|  | **M1** | **M2** | **M3** | **M4** |
| **Membrane surface area (m^2^)** | 0.24 | 0.5 | 0.76 (0.38 each) | 0.86 (0.43 each) |
| **Pore size (µm)** | 0.4 | 0.1 | 0.1 | 0.1 |
| **Outside diameter (mm)** | 25 | 25 | 25 | 41 |
| **Number of channels** | 19 | 61 | 19 | 19 |
| **Chanel diameter (mm)** | 3.5 | 2.2 | 2.8 | 6 |
| **Length specific membrane area (m^2^/m)** | 0.20 | - | - | 0.358 |
| **Total length (mm)** | 1178 | 1178 | 1178 | 1200 |
| **Maximum flow (L/h)** | 3600 | - | - | 9670 |
| **Hydraulic flow (mm)** | - | 2.2 | 2.8 | 3.87 |

1. **Figures**

**
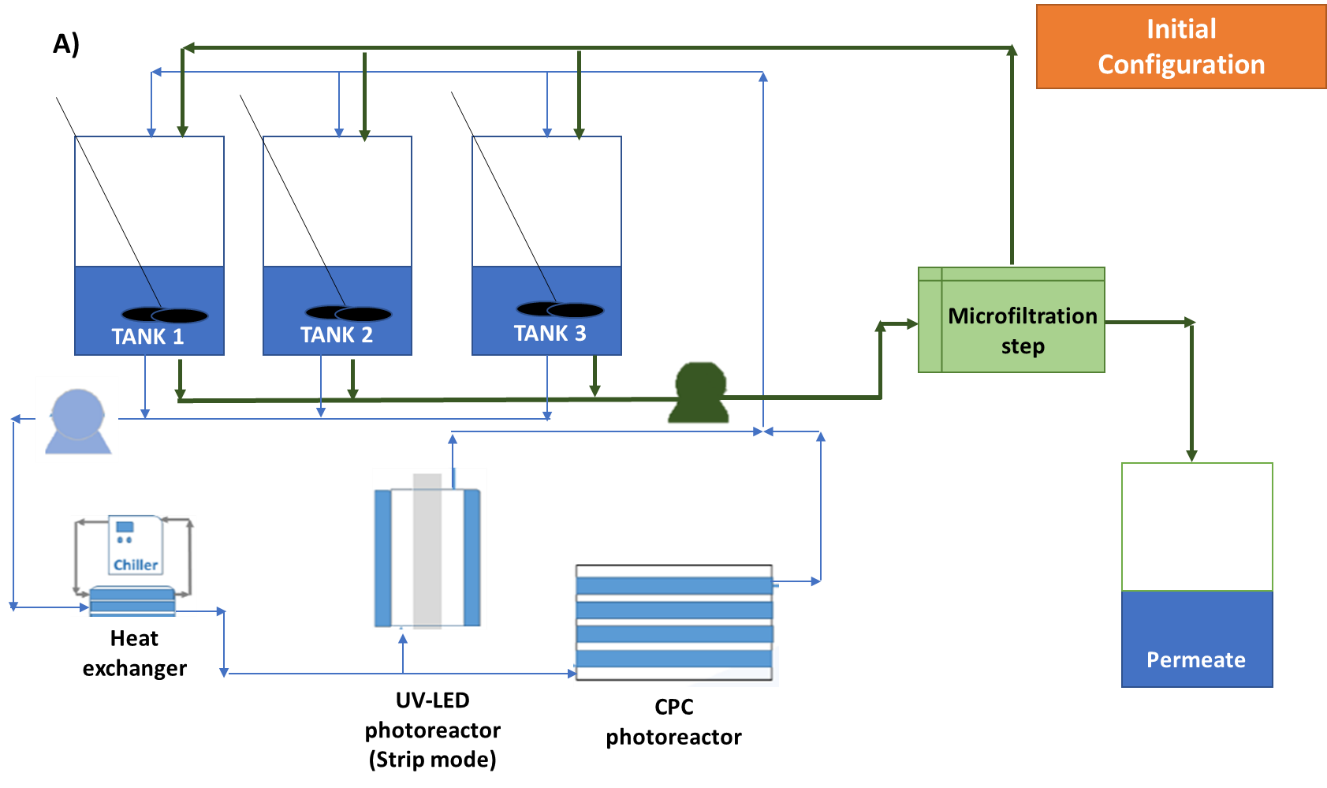
**


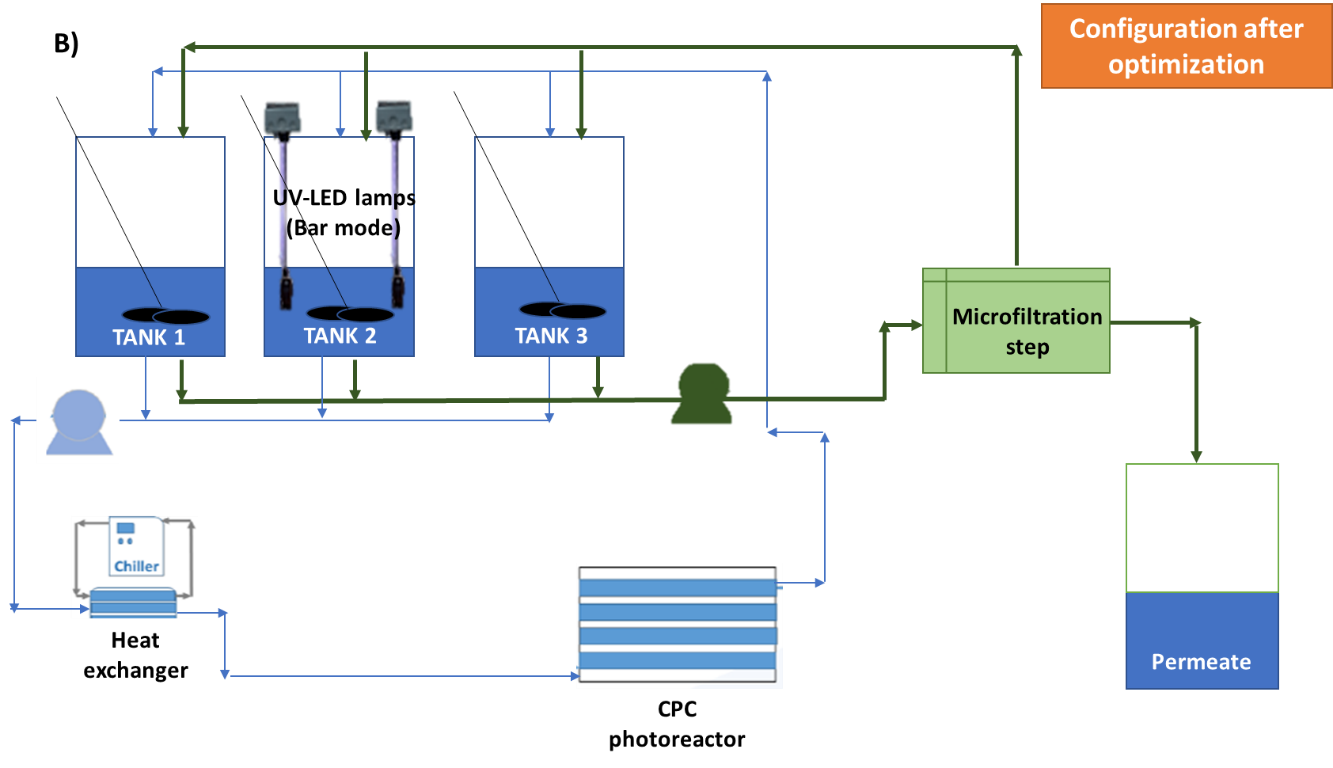


**Fig. S1.** Hydraulic scheme of the proposed tertiary treatment: a) initial configuration; b) configuration after optimization.

A)

B)


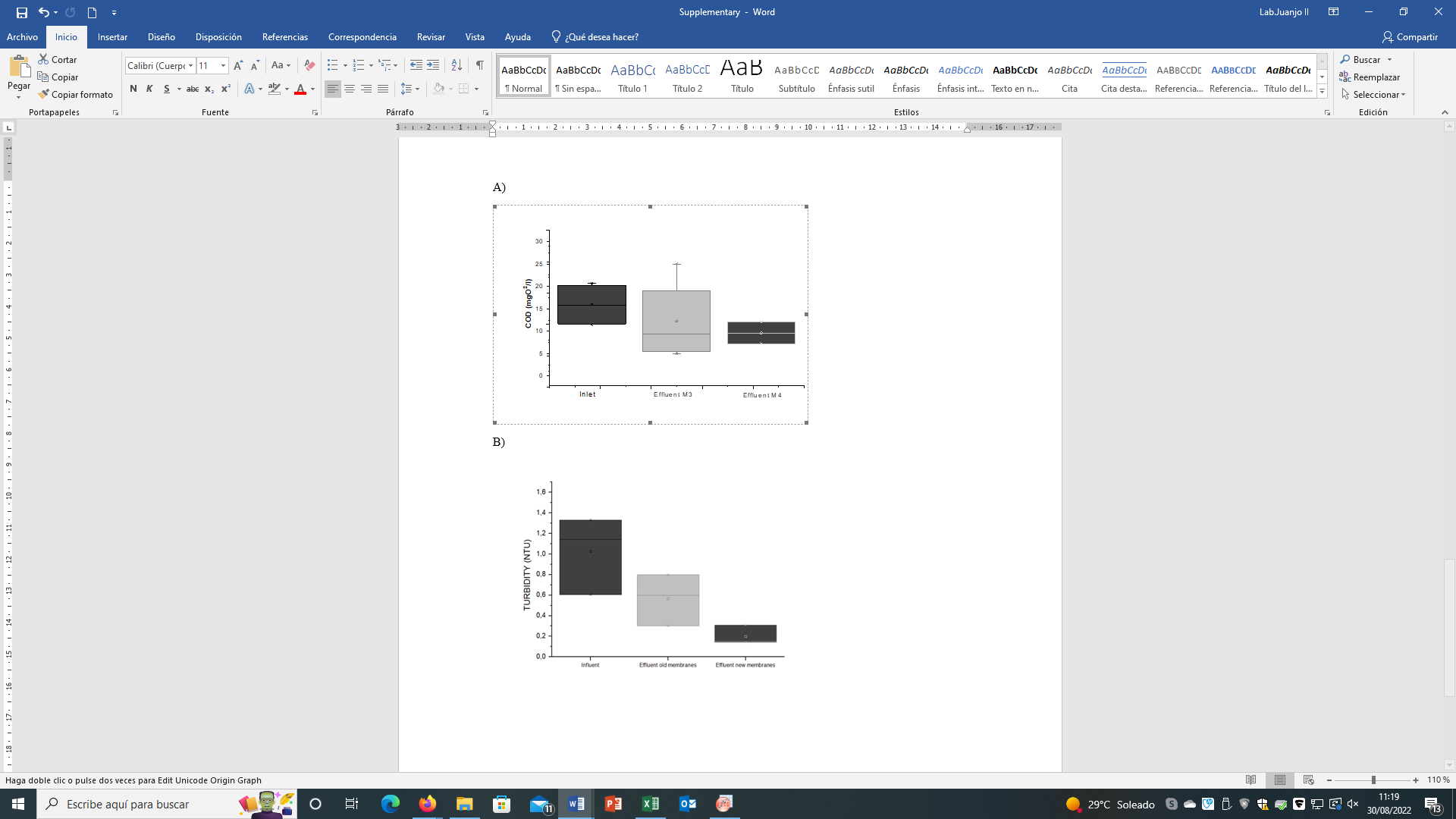

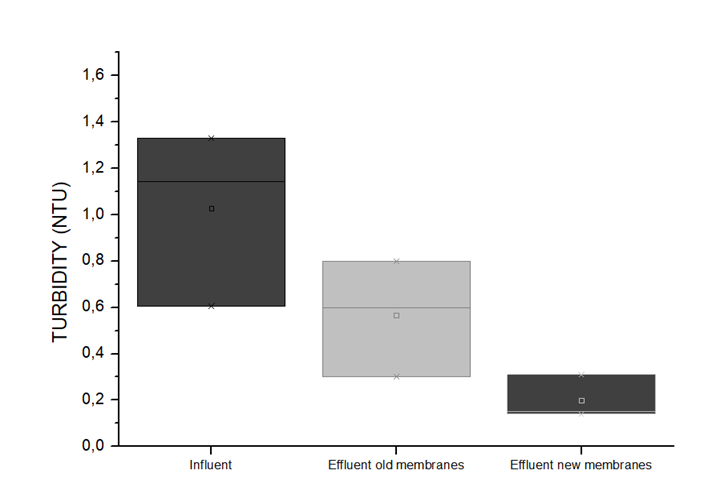


**Fig. S2.** COD and Turbidity removal efficiencies using M3 and M4 as a final filtration step.
